# Final results from a multicenter prospective study ( JROSG 05–5) on postoperative radiotherapy for patients with ductal carcinoma *in situ* with an involved surgical margin or close margin widths of 1 mm or less

**DOI:** 10.1093/jrr/rrv034

**Published:** 2015-06-20

**Authors:** Naoto Shikama, Kenji Sekiguchi, Naoki Nakamura, Hiroshi Sekine, Yuko Nakayama, Kazufumi Imanaka, Takeshi Akiba, Masahiko Aoki, Yoshiomi Hatayama, Etsuyo Ogo, Yoshikazu Kagami, Miho Kawashima, Kumiko Karasawa

**Affiliations:** 1 Department of Radiation Oncology, Saitama Medical University, International Medical Center, 1397-1 Yamane, Hidaka-City, Saitama, 350-1298 Japan; 2 Department of Radiation Oncology, St Luke’s International Hospital, 9-1 Akashi-cho, Chuo-ku, Tokyo, 104-8560, Japan; 3 Department of Radiation Oncology, National Cancer Center East, 6-5-1 Kashiwanoha, Kashiwa-shi, Chiba, 277-8577, Japan; 4 Department of Radiology, The Jikei University Third Hospital, 4-11-1 Izumihonmachi, Komae-shi, Tokyo, 201-8601, Japan; 5 Department of Radiation Oncology, Kanagawa Cancer Center, 2-3-2 Nakao, Asahi-ku, Yokohama, Kanagawa, 241-8515, Japan; 6 Department of Radiology, Nishikobe Medical Center, 5-7-1 Kojidai, Nishi-ku, Kobe, Shogo, 651-2273, Japan; 7 Department of Radiation Oncology, Tokai University, 143 Shimokasuya, Isehara, Kanagawa, 259-1193, Japan; 8 Department of Radiology, Hirosaki University School of Medicine and Hospital, 53 Honmachi, Hirosaki, Aomori, 036-8563, Japan; 9 Department of Radiation Oncology, Kurume University Hospital, 67 Asahimachi, Kurume-shi, Fukuoka, 830-0011, Japan; 10 Department of Radiation Oncology, Showa University Hospital, 1-5-8 Hatanodai, Shinagawa-ku, Tokyo, 142-8666, Japan; 11 Department of Radiology, Dokkyo Medical University, Koshigaya Hospital, 2-1-50 Mimamikoshigaya, Koshigaya-shi, Saitama, 343-8555, Japan; 12 Research Center for Charged Particle Therapy, National Institute of Radiological Sciences, 4-9-1 Anagawa, Inage-ku, Chiba-shi, Chiba, 263-8555, Japan

**Keywords:** ductal carcinoma *in situ*, breast cancer, margin width, radiotherapy, breast conservation

## Abstract

This multicenter prospective study ( Japanese Radiation Oncology Study Group: JROSG 05-5) aimed to evaluate the effectiveness of postoperative radiotherapy (PORT) in patients with ductal carcinoma *in situ* (DCIS) with an involved surgical margin or close margin widths of ≤1 mm or less. PORT consisted of whole-breast irradiation (50 Gy in 25 fractions) followed by boost irradiation (10 Gy in 5 fractions). Eligibility criteria were as follows: (i) DCIS without an invasive carcinoma component, (ii) age between 20 and 80 years old, (iii) involved margin or close margin widths of ≤1 mm, (iv) refusal of re-resection, (v) performance status of 0–2, and (vi) written informed consent. The primary endpoint was ipsilateral breast tumor recurrence (IBTR), and secondary endpoints were overall survival (OS), relapse-free survival (RFS), recurrence patterns, and adverse events. A total of 37 patients from 12 institutions were enrolled from January 2007 to May 2009. The median follow-up time was 62 months (range, 28–85 months). The median pathological tumor size was 2.5 cm (range, 0.3–8.5 cm). Of the 37 patients, 21 had involved margins, and 16 had close margins. The 5-year IBTR, OS and RFS rates were 6% (95% confidence interval [CI]: 2–21), 97% (95% CI: 83–99) and 91% (95% CI: 77–97), respectively. Two patients developed local recurrence at the original site after 39 and 58 months. No severe adverse events were found. Our study suggests that this PORT regimen could be a treatment option for patients with DCIS with involved margin or close margin who don't desire re-resection.

## INTRODUCTION

Ductal carcinoma *in situ* (DCIS) arising from the breast represents an intraductal epithelial proliferation of malignant cells and is considered to be a non-obligate precursor of invasive cancer [1, 2] Mammography screening programs and high-resolution magnetic resonance imaging have changed the clinical presentation of DCIS [3, 4]. Approximately one-fifth of all screen-detected breast cancers are now DCIS [4, 5]. Several randomized clinical trials have demonstrated that postoperative radiotherapy (PORT) after partial resection decreases the risk of ipsilateral breast tumor recurrence (IBTR) [6–9]. Breast-conserving therapy, including partial resection followed by PORT, has been one of the current standards of care for DCIS [2, 10, 11]. However, these randomized trials have mainly included low-risk patients who underwent partial resection and achieved negative surgical margins. There has been little evidence supporting the use of breast-conserving therapy for patients with high-risk DCIS such as those with a positive surgical margin or a narrow distance between surgical margins and tumor cells.

An involved surgical margin has been thought as one of the adverse prognostic factors for IBTR after breast-conserving therapy [12, 13]. It was reported that patients with surgical margin widths of <1 mm could benefit from PORT; however, an 8-year IBTR rate after partial resection followed by PORT was ∼30% [14]. The previous retrospective studies included various PORT regimens such as whole-breast irradiation alone and in combination with boost irradiation. Few prospective studies have evaluated the effectiveness of PORT exclusively for the patients with DCIS with an involved surgical margin or close margin, and a standard PORT regimen hasn't been established yet. This multicenter prospective study ( Japanese Radiation Oncology Study Group: JROSG 05-5) aimed to evaluate the effectiveness of PORT in patients with DCIS with an involved surgical margin or close margin widths of **≤**1 mm.

## MATERIALS AND METHODS

This multicenter prospective study was conducted to evaluate the effectiveness of PORT consisting of tangential whole-breast irradiation (50 Gy in 25 fractions) using photon beams followed by boost irradiation (10 Gy in 5 fractions) of the tumor bed using electron beams for patients with DCIS with an involved surgical margin or close margin widths of **≤**1 mm. Patients were eligible for inclusion in the study if they: (i) had DCIS without an invasive carcinoma component, (ii) were between 20 and 80 years of age, (iii) were diagnosed as having an involved margin or close margin widths of **≤**1 mm after pathological evaluation using 5-mm thick specimens, (iv) refused re-resection, (v) had a performance status of 0–2, and (vi) provided written informed consent. Exclusion criteria were: (i) bilateral breast cancers, (ii) diffuse calcification on the pre-treatment images, (iii) multiple tumors, (iv) macroscopic residual tumor, (v) axillary lymph node metastases, (vi) past history of chest irradiation, (vii) collagen vascular disease, (viii) pregnancy, (ix) active double cancer, (x) mental disorders, (xi) uncontrolled diabetes, (xii) uncontrolled hypertension, and (xiii) cardiovascular disease.

### Surgical resection and pathological evaluation

All patients were treated with breast-conserving surgery. The partial breast resection was performed with the appropriate surgical margin of 1 or 2 cm from the macroscopic tumor extension. Thirty-two patients received the axillary sentinel lymph node biopsy, and five patients did not receive axillary dissention and/or biopsy. The pathological evaluation of resection samples was conducted using 5-mm thick slices. A specimen mammogram was not performed; nor was a central pathological review. An involved surgical margin was defined as tumor cells on the surgical edge, and a close surgical margin was defined as the distance between the tumor cells and surgical edge being **≤**1 mm. A number of surgical clips, which were useful guides for the boost irradiation, were located at each edge of the resection cavity. The routine application of surgical clips was not used in all cases.

### Radiotherapy

All patients underwent computed tomography (CT) for data acquisition for the radiation treatment planning. CT scanning was performed in the supine position, and no respiratory control was used. Three-dimensional conformal radiotherapy (3D-CRT) treatment-planning software was used for all patients. Whole-breast irradiation was conducted using the tangential field technique with 4- or 6-MV photon beams. Simulation planning was performed to minimize radiation doses to the organs at risk, and to modify homogeneous dose distribution to fit target volumes using a wedge filter. Beam weights, beam angles, and wedge angles were manually optimized. A total dose of 50 Gy in 25 fractions for whole-breast irradiation was defined at the reference point. The reference point was placed in the center of the radiation field or vicinity. The electron beam field size for boost irradiation of the tumor bed was determined according to surgical clips, the surgical cavity, pathological findings and/or the surgical scar. The boost irradiation was mainly planned using the radiation treatment-planning system, and appropriate electron beam energy was selected according to the depth of the tumor bed. The boost irradiation field was decided according to the pre-surgical images, surgical findings, final pathological reports, and/or surgical clips.

### Statistical analysis

The primary endpoint was the IBTR, and secondary endpoints were overall survival (OS), relapse-free survival (RFS), recurrence patterns, and adverse events. IBTR was defined as any recurrence including invasive carcinoma type and DCIS type in the ipsilateral irradiated breast. OS time was defined as the time from registration to death due to any causes. RFS time was defined as the time from registration to treatment failure (such as recurrence in the ipsilateral breast, axillary node, or at a distant site) or death due to any causes. Toxicities were evaluated according to the Common Terminology Criteria for Adverse Events (CTC-AE) version 3.0. The 5-year estimated IBTR rate was projected as 20%, and the lower threshold of the 5-year IBTR rate was set at 45%. It was estimated that a sample of 36 patients was required, with a one-sided alpha of 0.05 and a statistical power of 90%. Kaplan–Meier methods were used to estimate IBTR, OS and RFS. All enrolled patients were included in the primary endpoint assessment. The last follow-up date was 27 October 2014. Patients were followed up every 6 months for 5 years, then once per year by clinical examination with or without annually mammography. Statistical analyses were performed with JMP software version 10 (SAS Institute, Cary, NC).

## RESULTS

The protocol concept was accepted in October 2005, and the full protocol was accepted in August 2006 by the Japanese Radiation Oncology Study Group ( JROSG) Executive Committee (Institutional Review Board of Saitama Medical University: No. 06-077-1). A total of 37 patients from 12 institutions were enrolled from January 2007 to May 2009. Two patients were enrolled simultaneously at the end of the enrollment, and total of 37 patients were enrolled in this study. The median follow-up time was 62 months (range, 28–85 months). The median age was 52 years (range, 33–78 years), and the median pathological tumor size was 2.5 cm (range, 0.3–8.5 cm). Patient characteristics are shown in Table 1. Sixteen patients had close margins, and 21 had involved margins. All patients received PORT per-protocol, and no patient interrupted PORT. Fourteen (38%) patients received adjuvant hormonal therapy.

**Table 1.** Patient characteristics

	*n* (%)	
Age (years)		Median 52 (33–78)
30–39	3 (8)	
40–49	11 (30)	
50–59	14 (38)	
60–70	6 (16)	
>70	3 (8)	
Pathological diameter (cm)		Median 2.5 (0.3–8.5)
<1.9	15 (41)	
2–3.9	6 (16)	
4–5.9	7 (19)	
>6	9 (24)	
Estrogen receptor		
Positive	26 (70)	
Negative	7 (19)	
Unknown	4 (11)	
Progesterone receptor		
Positive	22 (59)	
Negative	11 (30)	
Unknown	4 (11)	
Margin status		
Close margin	16 (43)	
Involved margin	21 (57)	
Axillary surgery		
Sentinel biopsy	32 (86)	
None	5 (14)	

**Fig. 1.**
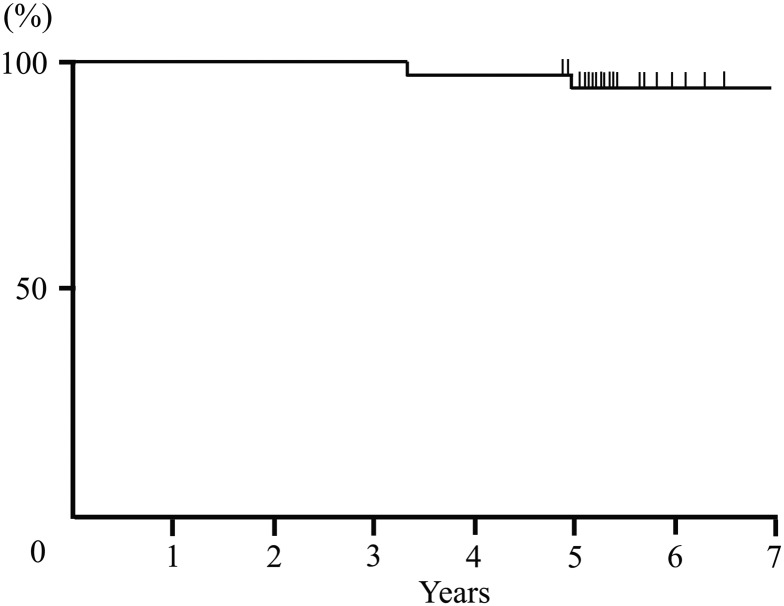
The ipsilateral breast tumor recurrence-free rate of all patients.

**Fig. 2.**
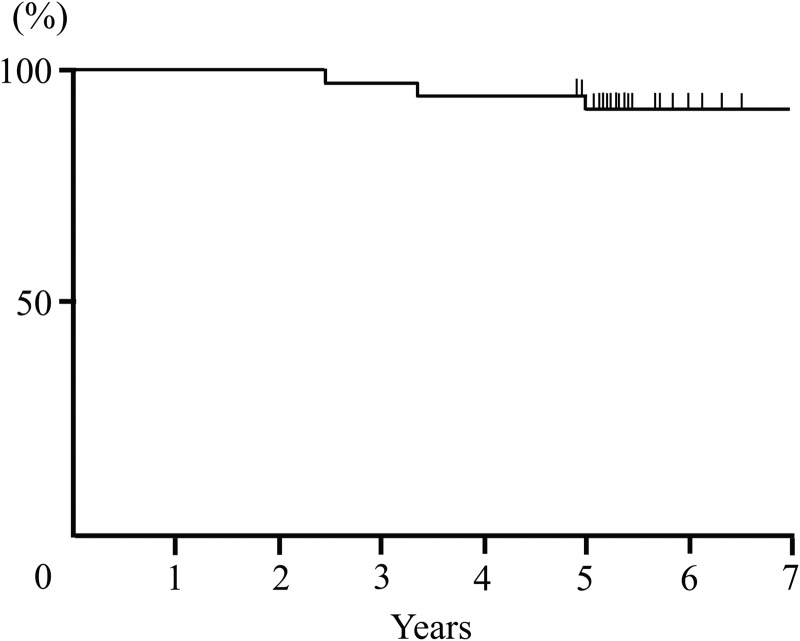
The relapse-free survival rate of all patients.

The 5-year IBTR, OS and RFS rates were 6% (95% confidence interval [CI]: 2–21), 97% (95% CI: 83–99) and 91% (95% CI: 77–97), respectively (Figs 1, 2). Two patients developed IBTR after PORT. One patient with an involved margin, who was 55 years old, developed IBTR at the original site after 39 months. The maximum diameter of the pathological tumor extension at the initial treatment was 8 cm. That patient underwent a salvage mastectomy, and the pathological diagnosis of IBTR was DCIS without an invasive carcinoma component. The other patient with IBTR was 45 years old, and she had a margin width <1 mm. She developed IBTR at the original site after 58 months. The maximum diameter of the pathological tumor extension at the initial treatment was 2.1 cm. She received salvage partial resection and axillary resection, and the pathological diagnosis of IBTR was DCIS. Although these two patients with IBTR had a positive estrogen receptor status, they didn't receive adjuvant hormonal therapy. They live without evidence of any more recurrence as at the time of the last follow-up. One patient died of colon cancer 28 months after registration, without experiencing breast cancer recurrence. No recurrence events were identified in regional lymph nodes or distant sites, and no severe adverse events (Grade 3 or 4) were reported as at the last follow-up.

## DISCUSSION

The current standard of care for patients with DCIS has consisted of mastectomy and breast-conserving therapy [1, 10]. A randomized controlled trial comparing mastectomy with breast-conserving therapy has not been performed, but current data demonstrate similar long-term survival times with either approach. Mastectomy has been mainly applied for patients with diffuse infiltrative disease, large tumors, or involved surgical margins after repeated resection [4]. Breast-conserving surgery followed by PORT is an acceptable treatment for a small and unifocal area of DCIS, and there is not enough evidence to omit PORT routinely. Several randomized control trials indicated that the administration of PORT after breast-conserving surgery reduced the IBTR rate by ∼60% [11, 15, 16]. The main goals of DCIS management are to reduce the risk of progression to invasive carcinoma, optimize breast cosmesis and prevent local recurrence [2, 17]. DCIS has a variable biological behavior, and there remains room for discussion regarding whether all patients with DCIS should be treated intensively. The impact of higher doses of radiotherapy in DCIS has been less clear, and Rakouitch *et al.* analyzed the data for 1895 patients from the population-based Ontario Cancer Registry and reported that the administration of boost irradiation after whole-breast irradiation was not associated with a lower IBTR [17]. On the other hand, the Rare Cancer Network conducted a multicenter retrospective study to evaluate the role of the administration of boost irradiation in patients 45 years or younger, and reported that compared with whole-breast irradiation alone, the administration of boost irradiation had a significant advantage (hazard ratio 0.45) [18]. There hasn't been enough evidence to confirm the role of administration of boost irradiation after whole-breast irradiation for DCIS, and the BONBIS trial in France has been now in ongoing.

Silverstein *et al.* developed a prognosis predictive model that included tumor size, margin width, and pathological classification (the Van Nuys Prognostic Index; VNPI) [12]. The patients with high VNPI scores showed high rates of IBTR after breast-conserving surgery followed by PORT. Investigators in the Memorial Sloan-Kettering Cancer Center developed a nomogram for predicting IBTR after breast-conserving surgery for DCIS using the data for 1868 consecutive patients [19]. Predictive factors consist of age, margin status, adjuvant endocrine therapy, PORT, and treatment period. The margin status was categorized as involved/close margin (≤2 mm), or negative margin. Dunne *et al.* conducted a systematic review and reported that a margin threshold of 2 mm seemed to be as good as a larger margin when breast-conserving surgery for DCIS was combined with PORT [20]. Wang *et al.* conducted a meta-analysis of the margin threshold for 7564 patients with DCIS [21]. This study demonstrated that the 10-mm threshold had the lowest odds ratio [OR] (patients with positive margins being the reference group) of IBTR (OR = 0.17) compared with the OR of IBTR for a 2-mm threshold (OR = 0.38), and that for a 5-mm threshold (OR = 0.55). On the other hand, Sahoo *et al.* analyzed the 103 consecutive patients with DCIS, and reported that involved margin status had a strong association with IBTR compared with close or negative margin status [22]. They reported that the 5-year IBTR was 7% in patients with a negative or close surgical margin as compared with 31% in patients with an involved margin.

Silverstein *et al.* reported that patients with tumor margin widths <1 mm could benefit from PORT, and an 8-year IBTR probability after breast-conserving surgery combined with PORT was 30% in comparison with 58% after breast-conserving surgery alone [14]. Approximately 80% of recurrence after breast-conserving surgery followed by PORT occurred within the first three years. Cutuli *et al.* conducted the multicenter retrospective analyses using 705 patients with DCIS treated between 1985 and 1995 in nine French regional cancer centers [23]. They reported that patients with negative, involved or uncertain margins had a 7-year crude IBTR rate of 9.7%, 25.2% and 12.2%, respectively. IBTR occurred within 5 years in 63.6% and 81.3% of patients receiving breast-conserving therapy combined with PORT versus breast-conserving surgery alone. They emphasized that the IBTR risk was higher in patients **≤**40 years of age among those with incomplete excision. These retrospective studies included various radiotherapy regimens consisting of whole-breast irradiation (40–50 Gy) combined with/without boost irradiation (10–20 Gy) to the tumor bed via brachytherapy or electron beam therapy. We conducted the prospective study for high-risk DCIS using whole-breast irradiation (50 Gy in 25 fractions) followed by boost irradiation (10 Gy in 5 fractions) via electron beam therapy, and decided that the primary endpoint was the 5-year IBTR rate. Our prospective study showed that the 5-year IBTR rate was only 6% after PORT, and it was indicated that this PORT regimen was a promising schedule for patients with a single DCIS lesion with an involved surgical margin or close margin widths of ≤1 mm.

Tamoxifen may be considered as an adjuvant endocrine treatment in patients with estrogen receptor–positive disease [1, 13]. Although the relative benefit of tamoxifen is ∼30–50%, the absolute reduction is only ∼2–4%, which may not justify the clinical benefits of endocrine treatment. There was no differential impact of Tamoxifen for patients with or without adverse pathological characteristics. The role of systemic treatments of DCIS needs further investigation [10, 13]. A number of ongoing studies are evaluating the effects of aromatase inhibitor and human-epithelial receptor-2 antibody. Further research focused on molecular and biological profiling is likely to enable personalized treatment strategies in order to minimize treatment harm [1, 10]. We did not evaluate the role of hormonal therapy because of the small sample size and unplanned subgroup analysis.

The limitations of this study are its small sample size and relatively short follow-up time. Silverstein *et al.* reported that ∼80% of recurrence after breast-conserving surgery followed by PORT occurred within the first three years [14], and then we decided the primary endpoint was the 5-year IBTR rate. However, our experience demonstrated that IBTR occurred after 3 years, and 5-year or longer follow-up duration is desirable. In addition, a central pathological review wasn't conducted. Although a central pathological review system was not established at the start of this trial, we determined that the pathological evaluation of resection samples would be conducted using 5-mm thick slices. We believed that this evaluation method provided accurate pathological evaluation of the tumor extension and margin width. Furthermore, symptomatic DCIS is associated with higher rates of IBTR compared with screen-detected disease [4, 10]. We did not collect the data concerning detection methods from the case report forms. We did not decide the post-surgical images including magnetic resonance images and mammograms, and did not collect the pathological data for focally surgical margin–positive versus diffuse surgical margin–positive.

## CONCLUSIONS

This prospective study suggests that this radiotherapy schedule (whole-breast irradiation followed by boost irradiation) could be a treatment option for patients with DCIS with an involved margin or a close margin who don't desire repeated surgery. A large-scale randomized trial is required, however, to make any definitive conclusions.

## FUNDING

Funding to pay the Open Access publication charges for this article was provided by the Japanese Radiation Oncology Study Group.
